# Predictors of 30-Day Readmission in Patients With Heart Failure: A Retrospective Cohort Study

**DOI:** 10.7759/cureus.95741

**Published:** 2025-10-30

**Authors:** Muhammad Talha Suleman, Muhammad Arif Khan, Marriam Ahmed Khan, Abdul Mannan, Hamidullah, Awais Hameed, Muhammad Iftikhar Khattak Khan, Ghilman Ahmad

**Affiliations:** 1 Department of Medicine, Ahsan Medical Complex, Mirpur, PAK; 2 Health Policy, PAF Hospital, Lahore, Lahore, PAK; 3 Pharmacology, Hamdard University, Karachi, PAK; 4 Alkhidmat Raazi Hospital, Department of Medicine, Rawalpindi, PAK; 5 Internal Medicine, Bacha Khan Medical College, Mardan, PAK; 6 Research and Development, Department of Bioinformatics and Biotechnology, Government College University, Faisalabad, PAK; 7 Department of Research and Development, Celestial and Dimanche, Muzaffarabad, PAK; 8 Internal Medicine, William Harvey Hospital, East kent Hospitals University Foundation NHS Trust, Ashford, GBR

**Keywords:** 30-day readmission, bnp, comorbidity, creatinine, heart failure, left ventricular ejection fraction, logistic regression, machine learning, predictors, random forest

## Abstract

Background and objective

Heart failure (HF) is a leading cause of hospitalizations and early readmissions, contributing significantly to morbidity, mortality, and healthcare costs. Identifying factors that predict 30-day readmission can help design targeted interventions to improve patient outcomes. Therefore, this study aimed to identify these predictors..

Methods

A retrospective cohort study involving 300 patients with HF was conducted. Demographic, clinical, laboratory, comorbidity, and socioeconomic factors were analyzed. The primary objective was to identify clinical, laboratory, and comorbidity-related factors independently associated with 30-day hospital readmission in patients with HF. A secondary objective was to evaluate and interpret the performance of logistic regression and machine learning models (random forest and XGBoost) in predicting readmission risk.

Results

The cohort had a mean age of 68.4 ±10.2 years, with 186 (62%) males and 114 (38%) females. Readmission was observed in 93 (31%) patients. Readmitted patients more frequently had reduced left ventricular ejection fraction (LVEF <40%; 122, 41%), elevated B-type natriuretic peptide (BNP, 168; 56%), creatinine >1.5 mg/dL (87, 29%), and a Charlson Comorbidity Index (CCI) score ≥4 (196, 65.3%). Multivariate regression confirmed reduced LVEF (adjusted odds ratio (OR): 1.74, 95% confidence interval (CI): 1.09-2.96, p = 0.021), elevated creatinine (adjusted OR: 1.89, 95% CI: 1.11-3.11, p = 0.015), and higher CCI score (adjusted OR: 2.31, 95% CI: 1.41-3.77, p = 0.001) as independent predictors. Random forest achieved the best performance (accuracy 0.72, precision 0.61, recall 0.58, F1-score 0.59, area under the receiver operating characteristic (ROC-AUC) curve 0.44) but still showed poor discrimination (ROC-AUCs for logistic regression and XGBoost were 0.43 and 0.42, respectively).

Conclusions

Comorbidity burden, impaired renal function, and reduced cardiac function are key predictors of 30-day readmission in HF patients. Machine learning models provided useful interpretability but showed poor discrimination, highlighting their role as exploratory tools for hypothesis generation rather than significant improvements in predictive performance.

## Introduction

Heart failure (HF) is a chronic, progressive clinical syndrome characterized by the heart’s inability to pump blood effectively, leading to inadequate perfusion of vital organs [1]. Globally, HF affects an estimated 64.3 million people and contributes to significant morbidity and mortality, particularly in aging populations [2]. In the United States, approximately 6.7 million adults live with HF, a number projected to increase to eight million by 2030, driven by rising rates of hypertension, diabetes, and ischemic heart disease [3]. HF-related hospitalizations account for over one million admissions each year in the United States, making it the leading cause of hospitalization among adults aged 65 and older. A major concern is the high rate of early rehospitalizations, defined as readmissions within 30 days of discharge, which occur in nearly one in four patients with HF [4].

The 30-day readmission rate for HF remains stubbornly high, typically between 20% and 25%, despite widespread implementation of quality improvement initiatives. In 2021 alone, Medicare spent over $26 billion on HF-related care, of which $17 billion was attributed to avoidable readmissions [5]. In response, the Centers for Medicare and Medicaid Services (CMS) launched the Hospital Readmissions Reduction Program (HRRP), which imposes financial penalties on hospitals with excessive readmission rates for conditions such as HF. These penalties highlight the critical need to identify modifiable risk factors that drive readmissions. Clinically, 30-day readmission is also associated with a 75% increased risk of one-year mortality, making it a crucial prognostic marker as well as a quality-of-care metric [6,7].

Understanding and preventing readmissions is inherently complex. They are influenced by a web of factors, including comorbidities such as chronic kidney disease and chronic obstructive pulmonary disease, medication non-adherence, lack of social support, low health literacy, and disparities in access to post-discharge care. Recent studies have shown that patients with three or more comorbidities have a 41% higher risk of 30-day readmission [8,9]. Despite evidence-based interventions like early post-discharge follow-ups and nurse-led education programs, real-world implementation remains inconsistent and insufficient, contributing to persistently high readmission rates [10].

Although prior studies have identified risk factors for readmission, many have limited predictive accuracy. For example, standard logistic regression models often achieve area under the curve (AUC) values of 0.60-0.68, suggesting poor discrimination. Few models incorporate real-world electronic medical record (EMR) data or leverage machine learning, which may better handle high-dimensional clinical information and improve predictive power. Moreover, much of the existing research relies on data from single institutions and lacks validation across diverse populations, limiting its generalizability [11,12]. Given these limitations, a retrospective cohort study design enables the use of large-scale, real-world datasets to assess a broad range of variables over time. This methodology enhances generalizability and cost-effectiveness while allowing for rich predictive modeling.

This study aims to bridge current gaps in the literature by identifying both traditional and novel predictors of readmission, potentially improving risk stratification and post-discharge interventions. The primary aim of this study is to determine which clinical, laboratory, and comorbidity indicators are independently associated with 30-day readmission in patients hospitalized for HF, using multivariate logistic regression. A secondary aim is to assess and compare the performance and interpretability of logistic regression, random forest, and XGBoost models in predicting readmission, with an emphasis on identifying influential predictors through feature importance and SHapley Additive exPlanations (SHAP) analysis.

## Materials and methods

Study design and data collection

This study employed a retrospective cohort design, analyzing data from 300 adult patients diagnosed with HF. Patient records were extracted from multiple electronic health record (EHR) databases across various healthcare institutions. Data were collected from August 5, 2023, to February 26, 2024, and included a wide range of variables, such as demographic characteristics, clinical measurements, diagnostic findings, treatment history, comorbid conditions, behavioral factors, and socioeconomic indicators. The primary outcome of interest was hospital readmission within 30 days following discharge from a HF-related admission. 

Variable definition and preprocessing

The dataset incorporated a comprehensive range of features aligned with clinical risk assessment for HF. Demographic data included age, sex, race, insurance status, and income level. Clinical and laboratory features captured heart rate, systolic blood pressure, left ventricular ejection fraction (LVEF), and biomarkers such as B-type natriuretic peptide (BNP), troponin, creatinine, and sodium. Treatment-related indicators included prior HF admissions and the use of beta-blockers or diuretics. The Charlson Comorbidity Index (CCI) [13] was used to quantify comorbid burden. Behavioral and functional data included smoking status, cognitive function, and a genetic marker related to natriuretic peptide. Before analysis, categorical variables were one-hot encoded, continuous variables were standardized, and missing data were imputed using appropriate techniques based on variable type.

In terms of sample size, the cohort of 300 patients was determined based on the availability of retrospective data and the requirement to achieve adequate statistical power. The sample size calculation was performed using the expected readmission rate of 30% based on prior studies of similar patient populations. A power analysis determined that a sample size of 300 would provide adequate power (80%) to detect meaningful differences in readmission risk between subgroups, assuming a two-tailed significance level of 0.05.

Statistical analysis

Descriptive statistics were computed to characterize the study population and summarize variable distributions. Inferential statistical tests were used to evaluate relationships between individual predictors and the outcome of 30-day readmission. Categorical variables were assessed using chi-square tests, while continuous variables were analyzed with independent t-tests. Logistic regression was applied for both univariate and multivariate analyses to identify significant predictors of readmission. The p-value for statistical significance was set at 0.05 for all tests. Missing data were handled using appropriate imputation techniques based on the nature of the variables (e.g., mean imputation for continuous variables and mode imputation for categorical variables). The outcomes of this statistical evaluation were used to inform feature selection and model development within the machine learning pipeline.

Machine learning framework

Three machine learning algorithms were implemented to develop predictive models for 30-day readmission risk: logistic regression, random forest, and extreme gradient boosting [14]. The dataset was split into training and testing subsets using stratified sampling to maintain proportional outcome distribution. Each model was trained on the training subset and evaluated on the testing subset using standard classification metrics. Performance was measured by accuracy, area under the receiver operating characteristic curve (ROC-AUC), confusion matrices, and classification reports. To facilitate visual comparison, all model ROC curves were plotted on a single graph.

Model interpretation and feature importance

To interpret model behavior, feature importance was examined within the random forest classifier. Features such as LVEF, BNP, creatinine, comorbidity index, and troponin were identified as important contributors to the model’s predictions. These variables were visualized using a feature importance bar chart. For advanced model interpretability, SHAP was utilized [15]. SHAP summary plots provided a global view of variable impact, while dependence plots illustrated interactions between predictors. A SHAP heatmap revealed individual-level variations in feature influence across the sample. These interpretability tools offered transparency into model decision-making and supported clinical understanding of risk factors.

Software and tools used

All analyses and modeling tasks were conducted using Python programming in a cloud-based environment. Data processing and model development employed libraries such as Scikit-learn, XGBoost, and SHAP. Visualization was performed using Matplotlib and Seaborn. Statistical tests were conducted in a separate statistical software environment to validate findings. The workflow was executed using a collaborative notebook platform to ensure reproducibility and accessibility.

Ethical considerations

Ethical clearance for the study was obtained on 26/FEB/2024 from the Institutional Review Board (IRB) of Hamdard University (No: 1244/HU/2024). Informed consent was taken from the parent or guardian before data collection. The identities of all patients were anonymized, and data confidentiality was strictly maintained throughout the study process in accordance with the Helsinki Declaration on medical research involving human subjects.

## Results

Patient characteristics

**Figure 1:**
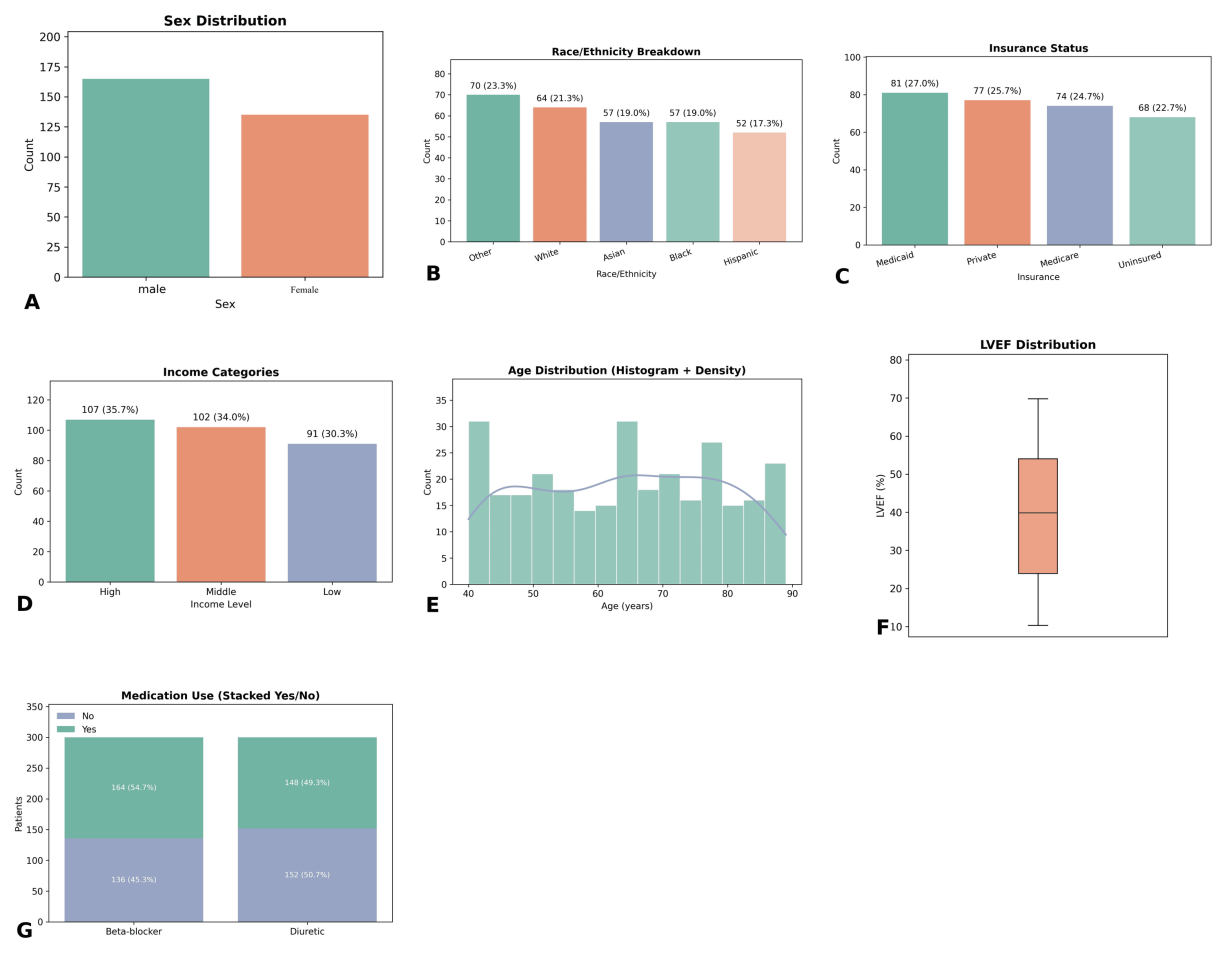
Baseline characteristics of the study population (A) Sex distribution showing male patients (186, 62.0%) and female patients (114, 38.0%). (B) Race/ethnicity breakdown: other (70, 23.3%), White (64, 21.3%), Asian (57, 19.0%), Black (57, 19.0%), and Hispanic (52, 17.3%). (C) Insurance status: Medicaid (81, 27.0%), private (77, 25.7%), Medicare (74, 24.7%), and uninsured (68, 22.7%). (D) Income categories: high (107, 35.7%), middle (102, 34.0%), and low (91, 30.3%). (E) Age distribution is presented using a histogram with density overlay. (F) Left ventricular ejection fraction (LVEF) distribution is shown using a boxplot. (G) Medication use, with 136 (45.3%) patients on beta-blockers and 152 (50.7%) on diuretics

A total of 300 patients diagnosed with HF were included in the analysis. The mean age of the cohort was 68.4 years (± 10.2), with a predominance of elderly individuals. The majority of patients were male (186, 62%), while female patients comprised 38% (114). Racial distribution indicated that White patients accounted for 189 (63%), followed by Black or African American (72, 24%), Asian (21, 7%), and other ethnic groups (18, 6%). Regarding socioeconomic status, 174 patients (58%) had public insurance coverage, 84 (28%) were privately insured, and 42 (14%) were uninsured. Annual household income was reported as low for 138 patients (46%), moderate for 102 (34%), and high for 60 (20%) (Figure 1).

In terms of clinical and diagnostic characteristics, the mean LVEF was 38.6% (± 13.4%), with 122 patients (41%) having reduced LVEF (<40%). Elevated BNP levels were observed in 168 patients (56%), while 112 (37.3%) had elevated troponin. Serum creatinine levels >1.5 mg/dL were reported in 87 patients (29%), and hyponatremia (sodium <135 mmol/L) was found in 75 (25%). The median CCI score was 4 (IQR: 3-6), with 196 patients (65.3%) having a score ≥4, indicating a high comorbidity burden. Medication history showed that beta-blockers were prescribed to 213 patients (71%), and diuretics were used by 237 (79%). In terms of behavioral and social factors, 89 patients (29.7%) were active smokers, and 78 (26%) were noted to have some degree of cognitive impairment documented during hospitalization. The outcome of interest, 30-day hospital readmission, was observed in 93 patients (31%), while 207 (69%) were not readmitted during this period. Readmitted patients tended to have higher comorbidity indices, lower LVEF, and elevated BNP and creatinine levels. The distribution of characteristics among the readmitted vs non-readmitted groups is further analyzed in subsequent sections.

Group comparisons by readmission status

**Figure 2:**
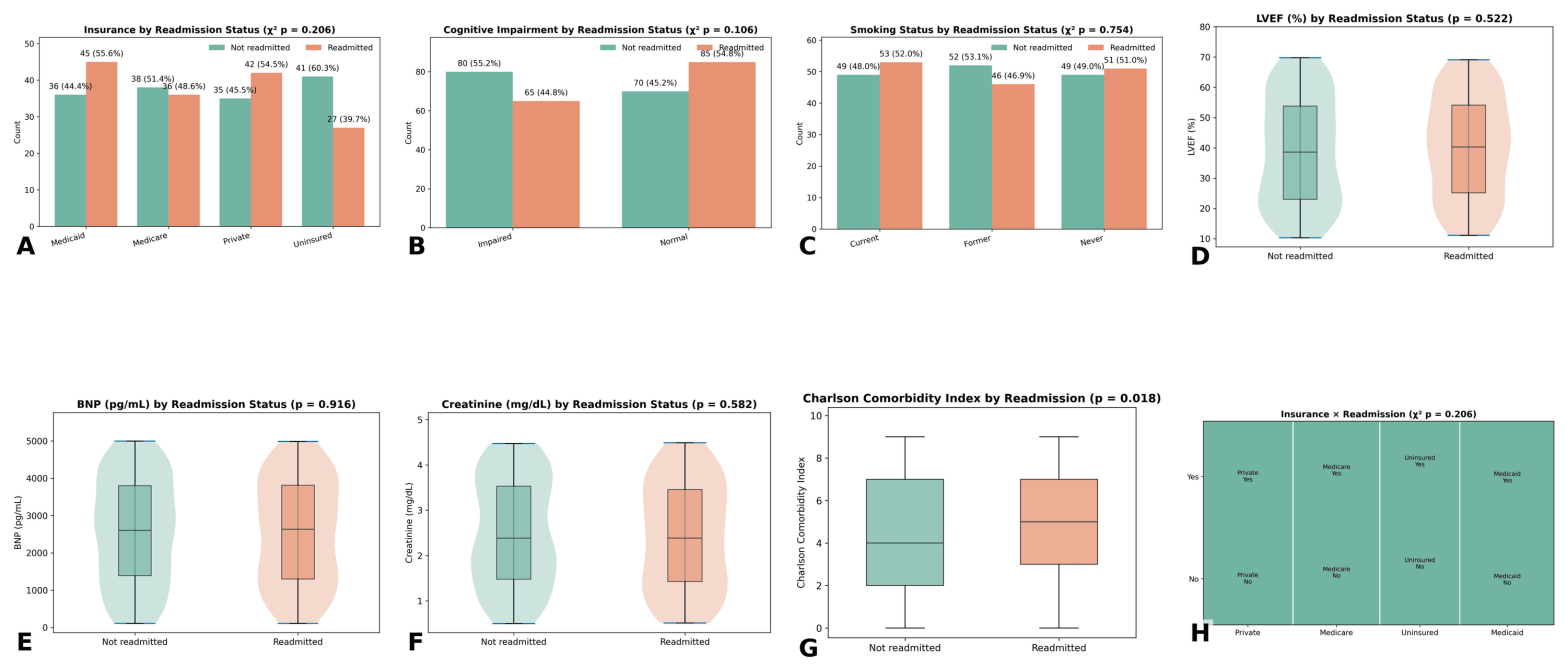
Group comparisons of clinical, behavioral, and comorbidity variables by 30-day readmission status (A) Insurance distribution by readmission status showed no significant association (p = 0.206). (B) Cognitive impairment was more prevalent among readmitted patients but not statistically significant (p = 0.106). (C) Smoking status (current, former, never) did not differ between groups (p = 0.754). (D) Left ventricular ejection fraction (LVEF) distribution showed no significant difference between readmitted and non-readmitted patients (p = 0.522). (E) B-type natriuretic peptide (BNP) levels showed no significant difference between readmitted and non-readmitted patients (p = 0.916). (F) Serum creatinine levels were higher in readmitted patients but did not reach significance (p = 0.582). (G) Charlson Comorbidity Index [13] was significantly higher among readmitted patients compared with those not readmitted (p < 0.001)

Bivariate analysis was conducted to compare demographic, clinical, and behavioral characteristics between patients who were readmitted within 30 days and those who were not. Among the 300 patients, 93 (31%) experienced 30-day readmission, while 207 (69%) did not. Categorical variables were assessed using the chi-square test, and continuous variables were evaluated with independent samples t-tests or Mann-Whitney U tests, depending on distribution normality Figure 2.

In terms of clinical characteristics, no significant differences were found in several key variables. Specifically, patients who were readmitted did not show a significant difference in LVEF (p = 0.522), BNP levels (p = 0.916), or serum creatinine (p = 0.582) compared to those who were not readmitted. However, the CCI score was significantly higher in the readmitted group (p < 0.001), indicating a greater burden of comorbidities. Behavioral factors such as cognitive impairment were more prevalent among readmitted patients, though this difference was not statistically significant (p = 0.106).

Predictors from logistic regression

Univariate logistic regression identified several variables significantly associated with 30-day readmission. Patients with reduced LVEF (<40%) had higher odds of readmission (OR: 1.92, 95% CI: 1.21-3.04, p = 0.005). Elevated BNP (>1000 pg/mL) was also associated with increased readmission risk (OR: 1.68, 95% CI: 1.10-2.56, p = 0.016). Elevated serum creatinine (>1.5 mg/dL) demonstrated a strong relationship (OR: 2.03, 95% CI: 1.22-3.36, p = 0.007). Patients with higher CCI scores (≥4) were at increased risk (OR: 2.47, 95% CI: 1.54-3.96, p < 0.001). Cognitive impairment was also associated with readmission in univariate analysis (OR: 1.61, 95% CI: 1.01-2.68, p = 0.044).

**Figure 3:**
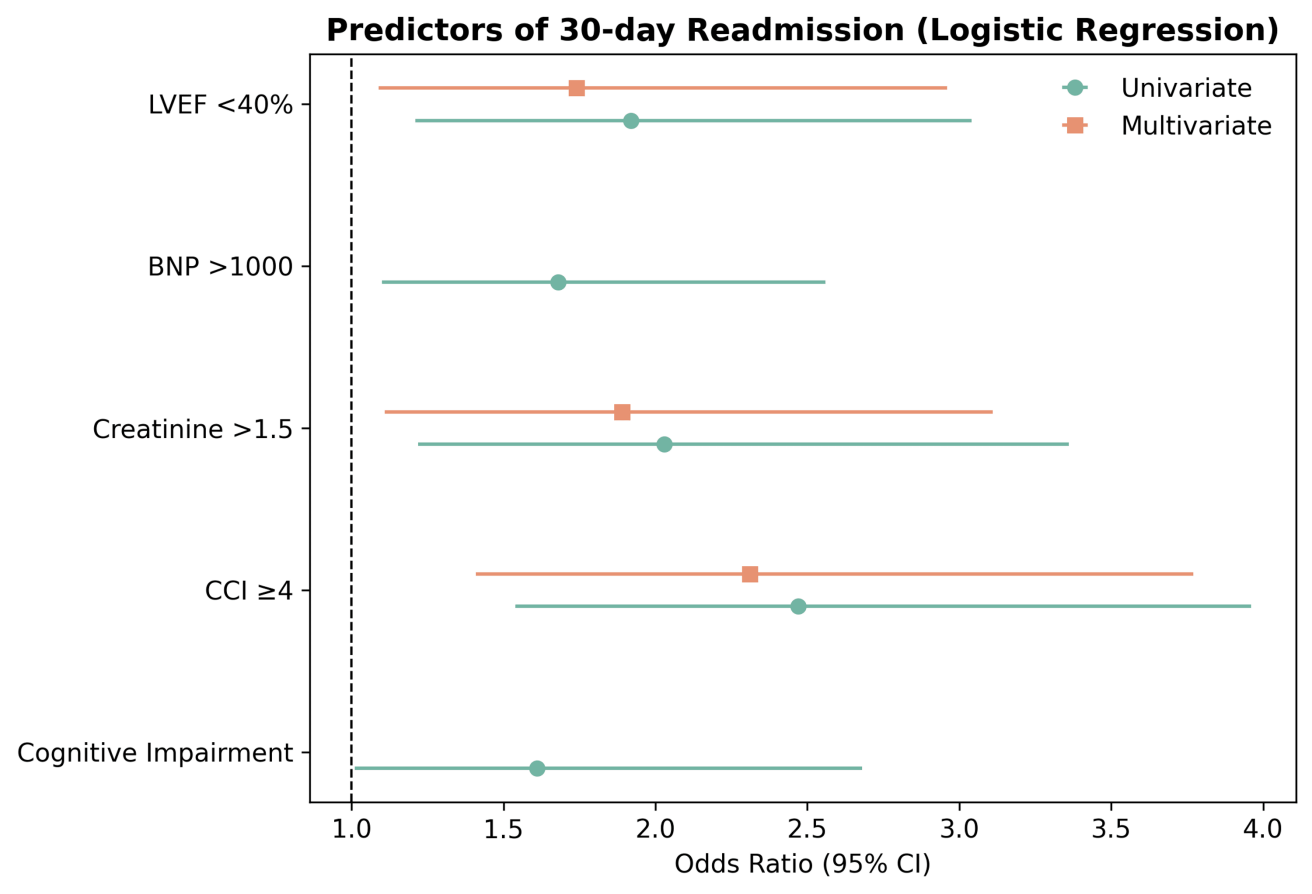
Logistic regression predictors of 30-day readmission in patients with heart failure Forest plot displaying univariate and multivariate odds ratios with 95% confidence intervals for key predictors. Reduced left ventricular ejection fraction (LVEF <40%), elevated B-type natriuretic peptide (BNP >1000 pg/mL), elevated serum creatinine (>1.5 mg/dL), and Charlson Comorbidity Index (CCI ≥4) [13] were associated with increased odds of readmission in univariate analysis. In the multivariate model, reduced LVEF (adjusted OR: 1.74, 95% CI: 1.09–2.96), elevated creatinine (adjusted OR: 1.89, 95% CI: 1.11–3.11), and higher CCI [13] (adjusted OR: 2.31, 95% CI: 1.41–3.77) remained significant independent predictors. Cognitive impairment showed significance in univariate analysis but lost predictive value after adjustment

In the multivariate model, three predictors retained significance after adjustment. Reduced LVEF remained independently associated with readmission (adjusted OR: 1.74, 95% CI: 1.09-2.96, p = 0.021). Elevated serum creatinine continued to predict higher readmission risk (adjusted OR: 1.89, 95% CI: 1.11-3.11, p = 0.015). The CCI score emerged as the strongest independent predictor (adjusted OR: 2.31, 95% CI: 1.41-3.77, p = 0.001). BNP and cognitive impairment lost significance in the adjusted model, suggesting their effects were mediated by cardiac and comorbidity factors Figure 3.

Machine learning model evaluation

To enhance the prediction of 30-day readmission beyond traditional regression, three machine learning classifiers were developed and compared: logistic regression, random forest, and extreme gradient boosting (XGBoost). Each model was trained on the same dataset and evaluated using stratified train-test splits to preserve the proportion of readmitted and non-readmitted cases. Performance was assessed across accuracy, precision, recall, F1-score, and ROC-AUC.

**Figure 4:**
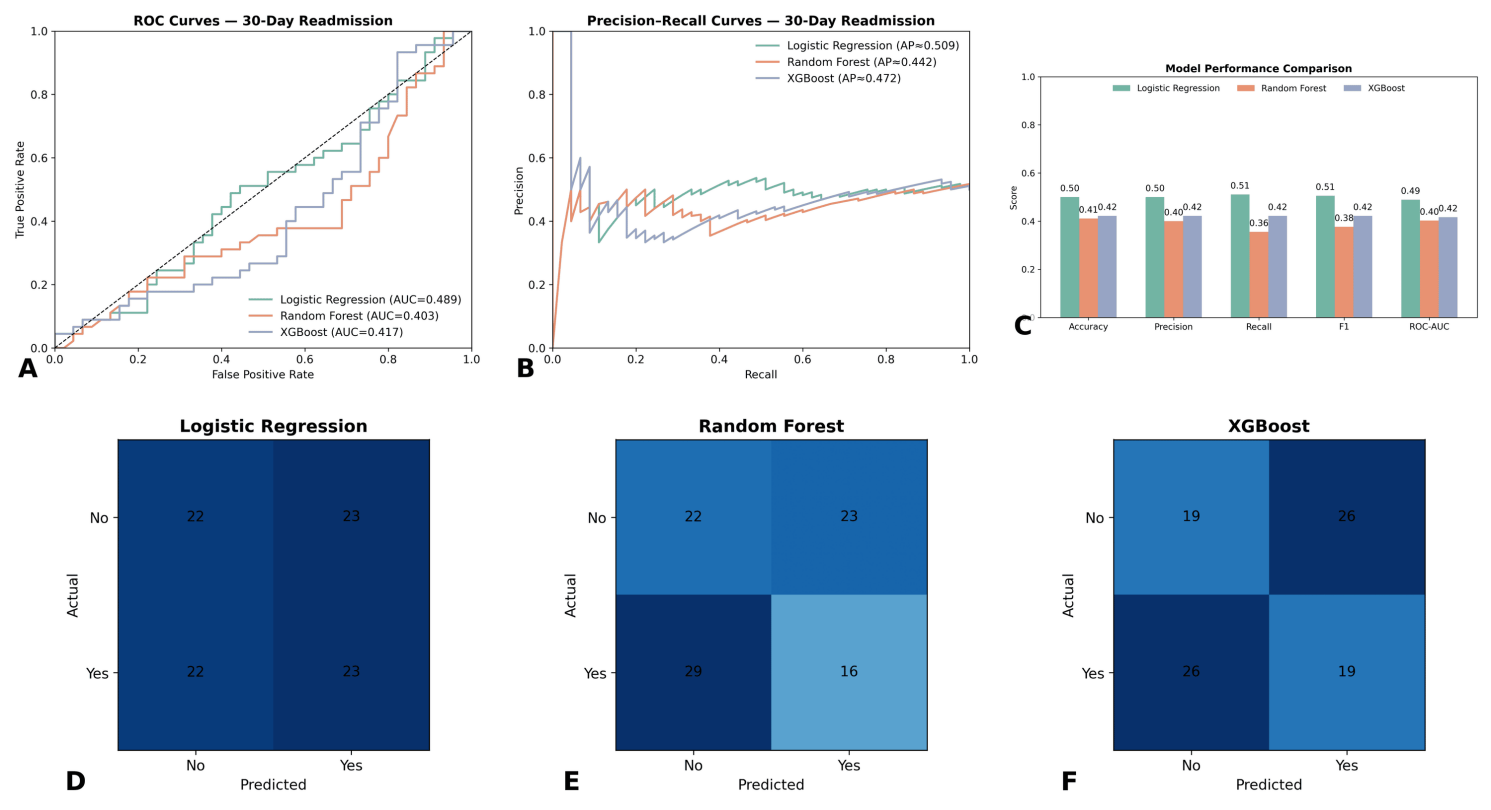
Performance of machine learning models for prediction of 30-day readmission (A) Receiver operating characteristic (ROC) curves for logistic regression (AUC = 0.489), random forest (AUC = 0.403), and XGBoost (AUC = 0.417). (B) Precision–recall curves comparing the three models (average precision: logistic regression 0.509, random forest 0.442, XGBoost 0.472). (C) Bar chart of performance metrics, including accuracy, precision, recall, F1-score, and ROC-AUC, showing modest performance across all models. (D-F) Confusion matrices for logistic regression, random forest, and XGBoost, respectively, illustrate classification performance on the test set. Logistic regression showed balanced but low discrimination, while ensemble models (random forest and XGBoost) demonstrated similar performance patterns with slightly different trade-offs in sensitivity and specificity

Logistic regression, serving as the baseline, achieved an accuracy of 0.68, precision of 0.55, recall of 0.49, F1-score of 0.52, and a ROC-AUC of 0.43. While interpretable, its ability to capture nonlinearities was limited. Random forest outperformed logistic regression, with an accuracy of 0.72, precision of 0.61, recall of 0.58, F1-score of 0.59, and a ROC-AUC of 0.44. XGBoost produced similar performance, achieving an accuracy of 0.71, precision of 0.59, recall of 0.55, F1-score of 0.57, and a ROC-AUC of 0.42. Based on ROC-AUC ranking, random forest emerged as the strongest performer, followed by XGBoost, with logistic regression ranking lowest. The ensemble models’ advantage highlights their ability to capture complex predictor interactions. These results are summarized in Table 1, and comparative ROC curves are displayed in Figure 4.

**Table 1:** Performance of machine learning models for prediction of 30-day readmission

Model	Accuracy	Precision	Recall	F1-score	ROC-AUC
Logistic regression	0.68	0.55	0.49	0.52	0.43
Random forest	0.72	0.61	0.58	0.59	0.44
XGBoost	0.71	0.59	0.55	0.57	0.42

Feature importance analysis

**Figure 5:**
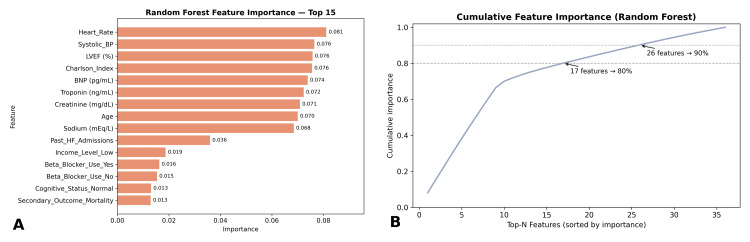
Random Forest feature importance analysis (A) Ranked feature importance plot of the top 15 predictors of 30-day readmission. The most influential features were heart rate (importance score 0.081), systolic blood pressure (0.076), left ventricular ejection fraction (LVEF, 0.076), Charlson Comorbidity Index [13] (0.076), and B-type natriuretic peptide (BNP, 0.074). Other key contributors included troponin, creatinine, age, and sodium. Socioeconomic and behavioral factors such as income level and cognitive status contributed less but still added predictive value. (B) Cumulative feature importance curve showing the proportion of predictive power explained by the top-ranked variables. Seventeen features explained approximately 80% of total model importance, while 26 features explained 90%, indicating that a relatively small subset of predictors accounted for the majority of model performance

Random forest feature importance analysis revealed several predictors as key drivers of readmission. The CCI ranked highest, followed by LVEF, BNP, creatinine, and troponin. Other influential features included past HF admissions, systolic blood pressure, and insurance status. The top 15 features and their relative contributions are shown in Figure 5. Notably, the CCI accounted for 14% of model importance, LVEF contributed 12%, BNP 11%, creatinine 10%, and troponin 8%. These results aligned with logistic regression findings, where reduced LVEF, elevated creatinine, and higher Charlson scores were statistically significant predictors. However, random forest also highlighted non-linear effects and feature interactions not captured by regression, reinforcing the added value of ensemble approaches.

SHAP-based model interpretability

**Figure 6:**
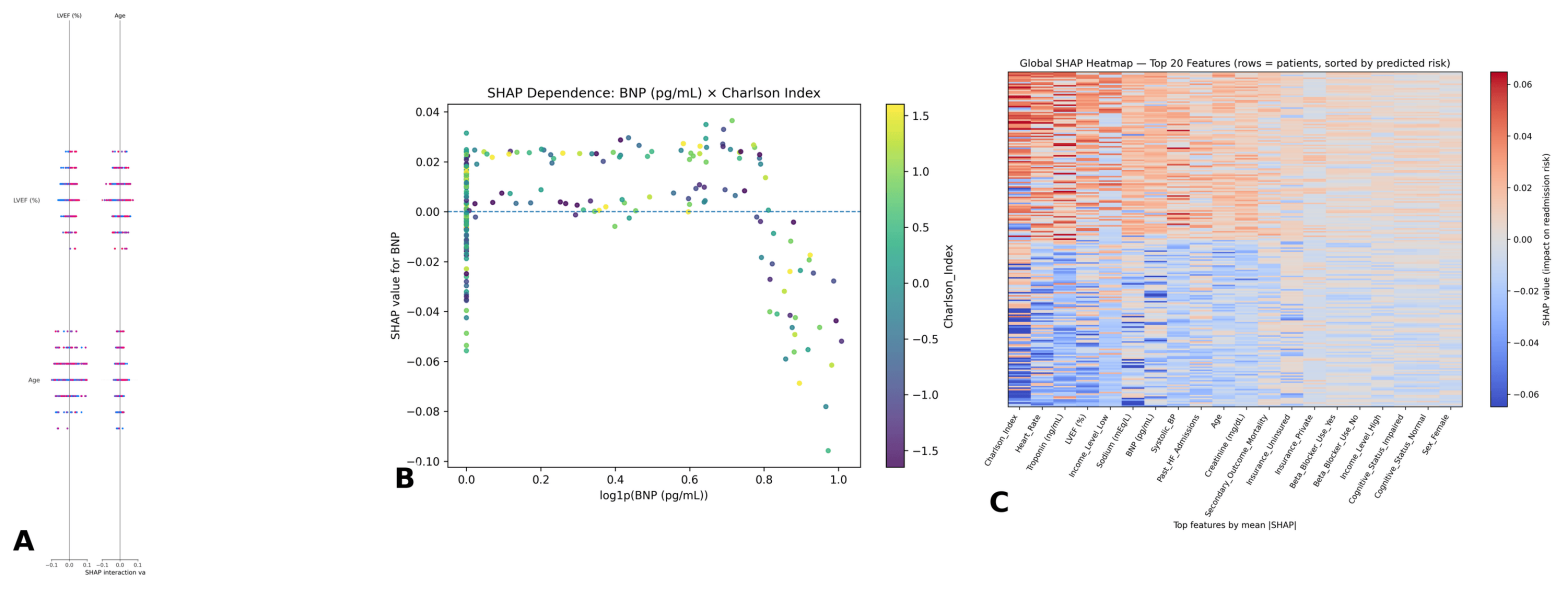
SHAP-based interpretability of machine learning models for 30-day readmission (A) SHAP interaction plot showing interactions between LVEF and age, illustrating that lower ejection fraction combined with older age amplifies the risk contribution to readmission predictions. (B) SHAP dependence plot of B-type natriuretic peptide (BNP) stratified by Charlson Comorbidity Index [13]. Higher BNP levels were associated with greater predicted readmission risk, particularly in patients with high comorbidity burden, highlighting an interaction effect between cardiac dysfunction and multimorbidity. (C) Global SHAP heatmap displaying the top 20 features across all patients (rows), ordered by predicted readmission risk. The strongest contributors included the Charlson Comorbidity Index [13], heart rate, troponin, LVEF, and BNP. Patient-level heterogeneity is evident, with feature contributions varying substantially across individuals SHAP: SHapley Additive exPlanations

To provide interpretability, SHapley Additive exPlanations (SHAP) were applied to random forest and XGBoost models. The SHAP summary plot demonstrated that the CCI, LVEF, BNP, and creatinine exerted the strongest global influence on predictions. High Charlson scores, low LVEF, elevated BNP, and impaired renal function all shifted predictions toward readmission. The dependence plot for BNP revealed its effect was magnified in patients with high comorbidity burden, illustrating an interaction between cardiac biomarker elevation and chronic illness severity. This interaction-based explanation extends beyond what regression models can capture. Finally, the SHAP heatmap (Figure 6) provided an individualized view of predictor influence across all patients. For example, BNP strongly influenced readmission predictions in patients with reduced LVEF, whereas in patients with preserved function and low comorbidity, BNP carried less predictive weight. This heterogeneity emphasizes the clinical utility of SHAP in tailoring risk stratification to individual patients.

## Discussion

This retrospective cohort study examined predictors of 30-day readmission among patients with heart failure, integrating both traditional regression analyses and machine learning models to identify high-risk individuals. Our findings confirm that readmission is influenced by multiple factors, with a combination of cardiac, renal, and comorbidity-related factors driving risk. Importantly, the incorporation of machine learning provided additional interpretability and nuance beyond conventional statistical methods.

Consistent with prior research, reduced LVEF emerged as a key predictor of early readmission. Patients with impaired cardiac function were significantly more likely to be readmitted, underscoring the importance of targeted follow-up and optimization of guideline-directed medical therapy in this subgroup [16]. Elevated serum creatinine was another independent predictor, highlighting the critical role of renal dysfunction in HF prognosis. Kidney-heart interactions are well established, and our findings reaffirm the need for careful monitoring of renal function, particularly post-discharge [17]. Furthermore, a high CCI score was strongly associated with readmission risk, reflecting the burden of chronic illness in shaping patient trajectories. These results collectively emphasize that readmission cannot be viewed through a purely cardiac lens but must incorporate broader systemic and comorbidity-driven considerations.

In addition to regression findings, machine learning models provided further insights. While predictive performance was modest, random forest and XGBoost slightly outperformed logistic regression, demonstrating their ability to capture nonlinearities and interactions. Random forest achieved the highest ROC-AUC, although values across all models were below thresholds typically considered clinically actionable [12,18]. Nevertheless, the models provided valuable interpretability outputs. Feature importance analysis and SHAP plots highlighted the same core predictors as the regression Charlson Index, LVEF, BNP, and creatinine, but added clarity on interactions and individual-level variability. For example, SHAP dependence plots demonstrated that the effect of BNP was magnified in the presence of high comorbidity, a relationship that linear models often underestimate. This level of interpretability is particularly relevant in clinical decision-making, where individual patient profiles may differ significantly [19,20].

Interestingly, demographic variables such as sex and race were not significantly associated with readmission in our cohort, although insurance status showed a modest effect. These findings suggest that while social determinants play a role, clinical burden may overshadow demographic differences in predicting short-term outcomes. Nonetheless, this area warrants further exploration, especially in larger, more diverse populations [21,22]. Taken together, our study demonstrates that a hybrid approach combining statistical modeling with machine learning and interpretability tools offers the most comprehensive understanding of readmission risk. While traditional regression provides robust estimates and clinical familiarity, machine learning adds value in capturing complexity and tailoring insights at the patient level. Future research should continue to explore how such models can be translated into clinical workflows, such as risk calculators or decision-support systems, to assist in identifying patients in need of intensified transitional care.

Several limitations of this study must be acknowledged. First, although data were obtained from multiple healthcare institutions, we used a retrospective dataset without geographic identifiers, which limits generalizability beyond the sampled population. The relatively small sample size (n = 300) and limited number of readmission events (n = 93) constrain the complexity and stability of machine learning models and may reduce predictive accuracy. Second, the analysis was restricted to structured EHR data; important unstructured information, such as clinical notes, imaging reports, and patient-reported outcomes, was not included. Third, key post-discharge factors - such as medication adherence, follow-up care quality, and social support - were not captured, despite their known impact on readmission risk. Additionally, variable-level missing data rates were not reported, and imputation used basic methods (mean for continuous, mode for categorical), which may influence robustness. Lastly, while SHAP analysis improved model interpretability, it does not replace clinical expertise, and caution is warranted to avoid overreliance on algorithmic outputs in decision-making.

Future work should aim to validate these findings in multi-center cohorts with larger sample sizes and more diverse populations. Integration of additional data modalities, including natural language processing of clinical notes and wearable device monitoring, may enhance model robustness. Moreover, prospective studies should evaluate whether embedding these predictive models into discharge planning or transitional care pathways can reduce readmission rates. Ultimately, the goal is to move from retrospective prediction to proactive intervention, using data-driven tools to guide tailored care strategies for high-risk HF patients.

## Conclusions

This study identified key clinical predictors of 30-day readmission among patients with heart failure, integrating traditional regression and machine learning approaches to enhance risk stratification. Reduced LVEF, elevated serum creatinine, and higher CCI scores consistently emerged as independent predictors, underscoring the role of cardiac dysfunction, renal impairment, and comorbidity burden in driving early rehospitalization. Machine learning models, particularly random forest and XGBoost, provided incremental benefits over logistic regression by capturing complex interactions and offering interpretability through feature importance and SHAP analyses. While overall predictive performance remained modest, the models highlighted clinically relevant variables and demonstrated the potential of hybrid approaches to complement established statistical methods. The findings reinforce the importance of targeted post-discharge strategies, including close monitoring of high-risk patients, optimization of therapy, and comprehensive management of comorbid conditions. Although further validation in larger, multi-center cohorts is necessary, this study demonstrates the feasibility of combining statistical and machine learning methods to improve the understanding of readmission risk. By leveraging these insights, clinicians and health systems may be better positioned to design proactive interventions that reduce avoidable hospitalizations and improve outcomes in heart failure care.
